# CD4^+^ T-lymphocytes exhibit biphasic kinetics post-myocardial infarction

**DOI:** 10.3389/fcvm.2022.992653

**Published:** 2022-08-25

**Authors:** Vinay Kumar, Sumanth D. Prabhu, Shyam S. Bansal

**Affiliations:** ^1^Department of Physiology and Cell Biology, The Ohio State University Wexner Medical Center, Columbus, OH, United States; ^2^The Dorothy M. Davis Heart and Lung Research Institute, The Ohio State University Wexner Medical Center, Columbus, OH, United States; ^3^Division of Cardiology, Department of Medicine, Washington University, St Louis, WA, United States; ^4^Division of Cardiovascular Medicine, Department of Internal Medicine, The Ohio State University Wexner Medical Center, Columbus, OH, United States

**Keywords:** myocardial infarction, T-cells, left-ventricular remodeling, heart failure, regulatory T-cells

## Abstract

CD4^+^ T-cells facilitate wound healing post-myocardial infarction (MI) but promote left-ventricular (LV) remodeling during ischemic heart failure (HF; 8 weeks post-MI). Therefore, it is critical to understand if sustained CD4^+^ T-cell activation leads to this pathological response, or if phenotypically different T-cells are activated during MI vs. HF. Using flow cytometry, we found that cardiac CD4^+^ T-cells exhibit two distinct patterns of transmigration. First pattern consisted of a rapid CD4^+^ T-cell response with maximal levels seen at 3 days post-MI which return to baseline by 14 days. However, during HF we observed a 2nd phase of activation and CD4^+^ T-cells were ∼20-fold higher in HF as compared to sham-operated mice. Importantly, these biphasic kinetics were observed with all major T-cell subsets such as Th1, Th2, Th17, and regulatory T-cells suggesting a global change. To determine the role of this 2nd peak of T-cell activation, CD4-iDTR mice were generated and treated with DT every 10 from 28 days post-MI to deplete CD4^+^ T-cells during chronic HF. While littermate control mice showed increased end-systolic and end-diastolic volumes (ESV and EDV) and decreased ejection fraction (EF) from 4 to 8 weeks post-MI, depletion of CD4^+^ T-cells in Cre + mice significantly blunted LV remodeling and inhibited progressive increases in the EDV and ESV, and reduction in EF. This suggests that CD4^+^ T-cell responses occurring during HF are different than those occurring during MI and promote LV remodeling and progressive cardiac dysfunction. Temporal immunomodulation of CD4^+^ T-cells could be a translatable modality for ischemic HF.

## Introduction

Sterile or non-sterile injury initiate a cascade of inflammatory immune responses required to repair the damaged tissue. Ischemic insult to the myocardium is no exception. Myocardial infarction (MI) induces a rapid and concerted response from different immune cells and inflammatory mediators with the objective of clearing dead and apoptotic cells, facilitate scar formation, and promote angiogenesis to restore tissue function and create a new, *albeit* altered, state of homeostasis (1). The innate immune response ensues rapidly (within min to hours) post-MI (1) and is mediated by neutrophils, monocytes, macrophages, and dendritic cells. Antigen processing by the phagocytic cells also lead to the activation of CD4^+^ (2) and CD8^+^ T-cells (3) in the mediastinal lymph nodes which then infiltrate the heart to aid innate immune responses and to contain overt immune activation (2).

Activation of CD4^+^ T-cells and its subsets is highly dynamic and follow complex patterns to coordinate tissue-clearance, scar formation and immune resolution post-MI. Studies conducted using global CD4^–/–^ mice have shown defects in myocardial healing with defective and disordered extracellular matrix deposition and increased mortality due to cardiac rupture suggesting their protective role during MI (2). However, irreparable damage caused during ischemia and limited regenerative potential of an adult mammalian heart often result in pathological LV remodeling and progressive cardiac dysfunction. Previous studies from us and others have shown that CD4^+^ T-cells are antigen-specific, display cardiac-memory and are essential drivers of left-ventricular (LV) remodeling during ischemic (4) and non-ischemic HF (5). Reasons for these contrasting findings regarding the role of CD4^+^ T-cells during MI vs. HF are unknown. It is possible that these pathological events could be a consequence of sustained T-cell activation either due to active antigen-presentation or a bystander phenomenon due to heightened pro-inflammatory cytokines/chemokines or a combination of both during HF (6). This will suggest that CD4^+^ T-cells undergo a phenotypic switch from being wound-healing during MI to pathological during HF, or different T-cells get activated during healing vs. LV remodeling post-MI. To answer this question, we conducted detailed kinetics of T-cell activation post-MI and measured cardiac levels of Th1 (IFNγ^+^), Th2 (IL-4^+^), Th17 (IL-17^+^), and Tregs (Foxp3^+^) at different time intervals. We also generated a new conditional CD4-iDTR mouse model to confirm the pathological role of CD4^+^ T-cells specifically during LV remodeling and chronic HF.

## Materials and methods

### Ethics statement, mouse model, and surgical protocol

Animal studies were conducted under an approved protocol by the Institutional Animal Care and Use Committee at the Ohio State University (IACUC protocol No. 2018A00000078R1) and were executed according to the NIH Guide for the Care and Use of Laboratory Animals (DHHS publication No. 85-23, revised 1996). Mice were kept in temperature and humidity-controlled vivarium with free access to food and water *ad libitum*. Male 10–12 week old C57BL/6 mice (WT) (Jackson, stock# 000664), CD4-Cre mice (Jackson Stock # 022071), and Rosa26-iDTR (inducible Diphtheria Toxin Receptor) mice (Jackson Strain # 007900) were used in these studies. Mice underwent either sham surgery or permanent left-anterior descending coronary artery ligation to induce MI or HF (8 weeks post-MI) under general anesthesia and mechanical ventilation, as described earlier (4). Peripheral blood (∼100 μL) was collected from the facial vein at different time intervals and processed for flow cytometric analysis, as described previously (7). After 8 weeks post MI or sham surgery, echocardiography was conducted to measure cardiac function followed by euthanasia to harvest heart for immune cell isolation.

### Cardiac digestion, immune cell isolation, and fixation

Leukocytes from the blood and the heart were isolated, fixed and stained using our previously published protocols (7). Briefly, hearts were minced using a heavy-duty single edge razor blade and transferred to a 50-mL conical tube containing 2-mL ice-cold PBS. Tubes were centrifuged at 50 g for 2 min to remove intravascular cells and 7–8 mL of digestion buffer containing 1 mg/mL of collagenase II (Worthington # LS004177) prepared in RPMI 1640 (Gibco) was added to each tube followed by incubation at 37°C for 20–25 min with gentle mixing every 5 min. 10 mL of cold autoMACS Running Buffer (Miltenyi Biotec # 130-091-221) was used to neutralize excess collagenase and digested contents were passed through 40-μm cell strainers (Fisher # 22-363-547). Small tissue chunks, if any, collected on the cell strainers were slowly triturated and 4 to 5 mL of digestion neutralization buffer was added onto the strainers to ensure complete inhibition of digestion enzyme. Cells were pelleted by centrifugation at 500 g for 8–10 min (4°C) and fixed using 1% PFA for 10 min on ice and excess fixing reagent was washed with cold PBS supplemented with 2% BSA and 2 mM EDTA. Fixed cardiac cell suspensions were stored at 4–8°C until stained for flow cytometry.

### Flow cytometric staining

Detailed staining protocols are described previously (7). Briefly, fixed single cell suspensions were incubated on ice with a cocktail of extra-cellular anti-mouse antibodies for 45 min. Cells were washed with cold PBS (supplemented with 2% BSA and 2 mM EDTA), permeabilized using 0.5% v/v tween-20 and incubated on ice with a cocktail of intra-cellular antibodies for 30 min. Finally, cells were washed with 4 mL of cold PBS and 2 μL of counting beads (Spherotech catalog#ACBP-100-10) were added to all the samples. Following antibodies were utilized in this study CD45-PEcy7 (Tonbo#60-0451-U100), CD45-Super Bright 600 (Invitrogen#63-0451-82), CD8-BV650 (BioLegend#100742), CD4-Super Bright 600 (Invitrogen#63-0041-82), CD4-PEcy7 (Invitrogen#63-0041-82), CD3-FITC (Tonbo#35-0032-U100), IFNγ- eFluor 450 (Invitrogen#48-7311-82), Foxp3-APC (Invitrogen#17-5773-82), IL4-PerCP-eFluor 710 (Invitrogen#46-7041-82), and IL17- Alexa Fluor 700 (BioLegend#506914).

### Echocardiography

Echocardiography was performed under 1–1.5% isoflurane using VisualSonics Vevo 3100 equipped with MX550D scan-head and adjustable heated rail system. Cardiac function was measured using parasternal B-mode images while maintaining a heart rate of > 480 bpm.

### Statistical analysis

All data are presented as mean ± SD. Statistical comparisons between 2-groups were performed using two-tailed paired or unpaired Student’s *t*-test with equal or unequal variance. Data representing more than 2 groups were analyzed using 2-way Analysis of Variance (ANOVA) with Bonferroni *post-hoc* test. GraphPad Prism version 9.3.1 was used to plot and analyze all data and a *p*-value < 0.05 was considered significant.

## Results

### CD4^+^ T-cells exhibit distinct infiltration patterns post-myocardial infarction and heart failure

**FIGURE 1:**
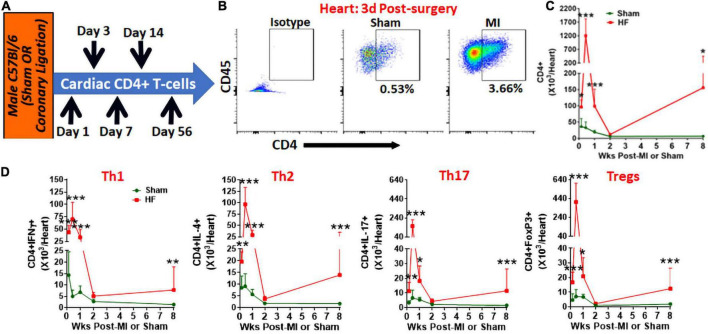
**(A)** Schematic for the experimental protocol to measure cardiac CD3^+^CD4^+^T-cells post-MI. **(B)** Representative flow cytometry plots for cardiac CD45^+^CD3^+^CD4^+^ T-cells in sham and MI mice at 3 days post-surgery, and **(C)** their group quantitation at 1 days, and 3 days, and 1-, 2-, and 8-weeks post-MI. **(D)** Group quantitation for cardiac Th1 (CD3^+^CD4^+^IFNγ^+^), Th2 (CD3^+^CD4^+^IL-4^+^), Th17 (CD3^+^CD4^+^IL-17^+^), and Tregs (CD3^+^CD4^+^FoxP3^+^) (8) at 1 day, and 3 days, and 1-, 2-, and 8-weeks post-MI. Data for 8 weeks has been shown previously (4). In the sham group *n* = 8, 5, 7, 5, and 7 whereas in the MI group *n* = 10, 5, 9, 5, and 10 at 1 day, 3 days, 1, 2, and 8-weeks, respectively, in **(C,D)**. Individual data points are not shown for clarity of trends. Data in **(C,D)** were analyzed using 2-way Anova with Bonferroni *post-hoc* test. *p < 0.05, **p < 0.01, and ***p < 0.001 represent significance with respect to indicated groups.

Using flow cytometry, we measured cardiac CD4^+^ T-cells at different time intervals post-MI or sham surgery (Figure 1A). As shown in Figures 1B,C, CD4^+^ T-cells rapidly infiltrated the myocardium and increased levels in the heart can be observed within 1 day post-MI (∼2.5-fold as compared to sham). CD4^+^ T-cell infiltration is maximal at 3 days post-MI with ∼40-fold higher numbers as compared to sham which drops down significantly by 7 days (∼4-fold) and are not different from sham mice by 14 days post-MI. Interestingly, these T-cell activation kinetics were similar to the kinetics seen with the innate immune cells (1) as the end of T-cell activation phase (2 weeks post-MI) coincided with the reported end of scar-formation phase (1) suggesting concerted activation of innate and adaptive immune responses post-MI. However, as the LV remodeling and cardiac dysfunction progressed, we again observed high levels of CD4^+^ T-cells (∼20-fold) as compared to sham by 8 weeks post-MI. These distinct patterns of T-cell activation during MI and HF were observed with all major helper T-cell subsets such as Th1, Th2, Th17 and regulatory T-cells (Tregs) (8) (Figure 1D). Comparison of Th1/Th2 ratios further showed an increased Th1 to Th2 ratio at 1 days post-MI as compared to sham (2.27 ± 0.68 vs. 1.63 ± 0.35 in sham) which was not different at later time-points. Similarly, Th17/Treg ratios were decreased at 1 days (0.698 ± 0.366) and 3 days (0.40 ± 0.082) post-MI as compared to sham (1.25 ± 0.67 and 1.16 ± 0.60, respectively) and were not different during HF.

### CD4^+^ T-cells are critical for progressive cardiac dysfunction and LV remodeling

**FIGURE 2:**
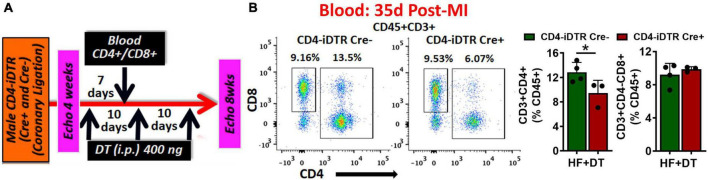
**(A)** Experimental plan for diphtheria toxin (DT) administration in CD4-iDTR Cre^+^ and Cre^–^ mice from 4 to 8 weeks post-MI. DT (400 ng, *i.p.*) was administered on days 28, 38, and 48 post-MI. **(B)** Representative flow cytometry plots for circulating CD4^+^ and CD8^+^ T-cells in CD4-iDTR Cre^–^ and CD4-iDTR Cre^+^ mice at 7 days post-injection (*i.p.*) of first DT dose (35 days post-MI) *(left)*, and their group quantitation *(right).* Data is shown from 1-representative experiment. Mean ± SD is shown. Unpaired 2-tailed Student’s *t*-test was used in **(B)**. *P < 0.05 represents significance with respect to the indicated groups.

**FIGURE 3:**
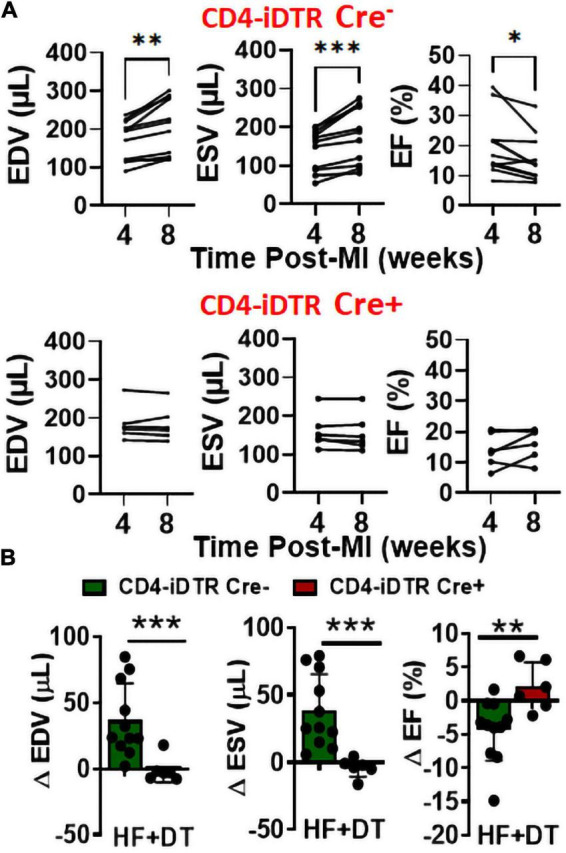
**(A)** End-diastolic and end-systolic volumes (EDV and ESV) and ejection fraction (EF) of CD4-iDTR Cre^–^ (littermate controls) and CD4-iDTR Cre^+^ mice at 4 weeks (during randomization) post-MI and at 8 weeks after treatment with three doses (repeated every 10 days) of 400 ng DT. **(B)** Change in the EDV, ESV, and EF of CD4-iDTR Cre^–^ (littermate controls) and CD4-iDTR Cre^+^ mice from 4 to 8 weeks post-MI after treatment with three doses (repeated every 10 days) of 400 ng DT. CD4-iDTR Cre^–^ mice were used as littermate controls and underwent similar DT injections. Mean ± SD is shown. Paired 2-tailed Student’s *t*-test was used in **(A)** whereas unpaired 2-tailed Student’s *t*-test was used in **(B)**. *p < 0.05, **p < 0.01, and ***p < 0.001 represent significance with respect to indicated groups.

Using a neutralizing anti-CD4 antibody (Gk1.5 clone), we previously showed that CD4^+^ T-cell activation during HF is pathological (4). However, this antibody is known to differentially affect CD4^+^ T-cells in young and aged mice (9). Therefore, to confirm the role of CD4^+^ T-cells during HF, we developed a transgenic mouse model by breeding CD4-Cre mice (Jackson Strain # 022071) with Rosa26-iDTR (inducible Diphtheria Toxin Receptor) mice (Jackson Strain # 007900) containing simian diphtheria toxin receptor (DTR) inserted into the ROSA26 locus. DTR expression in these mice is inhibited using an upstream loxP-flanked STOP codon. Thus, the stop sequence is deleted in homozygous (for iDTR gene) off-springs that are Cre^+^ resulting in the expression of DTR selectively in CD4^+^ T-cells to enable their reversible depletion upon DT administration at any time post-MI. Homozygous iDTR mice with or without Cre expression (Cre^+^ and Cre^–^) underwent coronary ligation. At 4 weeks post-MI mice were randomized according to their cardiac dysfunction and were treated with 400 ng of DT (*i.p.*) every 10 days. Detailed experimental plan is given in Figure 2A. At 35 days post-MI (7 days post-injection of first dose) we measured circulating levels of CD4^+^ and CD8^+^ T-cells. As shown in Figure 2B, DT administration significantly reduced CD4^+^ T-cells without affecting CD8^+^ T-cells. To determine the effect of T-cell depletion on cardiac function, we conducted echocardiography at 4- and 8-weeks post-MI. As shown in Figures 3A,B, CD4-iDTR Cre^–^ mice exhibited progressive increases in the end-diastolic and end-systolic volumes (EDV and ESV), and a decrease in the ejection fraction (EF) from 4 to 8 weeks post-MI. In contrast, DT mediated depletion of CD4^+^ T-cells in CD4-iDTR Cre^+^ mice completely blunted these changes and EDV and ESV, and EF did not worsen from 4 to 8 weeks post-MI.

## Discussion

While the role of innate immune responses in mediating wound healing and scar formation during MI (1) and pathological LV remodeling during chronic ischemic cardiomyopathy (2) are well characterized, role of CD4^+^ T-cell mediated adaptive immune responses is not clear. Early studies using whole body CD4^–/–^ mice showed that CD4^+^ T-cells are required for wound healing and adequate scarring (2). In contrast, our previous studies showed that CD4^+^ T-cells promote LV remodeling and progressive cardiac dysfunction during HF (4). Reasons for these discrepant results are not known. In this brief research report, we provide several novel findings to answer these long-standing questions. First, our data show two distinct patterns of CD4^+^ T-cell transmigration into the myocardium that is different during MI and chronic ischemic HF. Second, all major helper T-cell subsets, such as Th1, Th2, Th17 and Tregs, exhibited similar kinetics without any distinct pro- vs. anti-inflammatory phases, as has been demonstrated for innate immune responses (1). Third, using our CD4-iDTR mice we establish a pathological role of CD4^+^ T-cells during chronic HF and show that temporal depletion of CD4^+^ T-cells ameliorate LV remodeling and blunt progressive cardiac dysfunction.

MI induces rapid influx of CD4^+^ T-cells into the myocardium probably to regulate and assist innate immune responses in wound-healing and scar-formation and diminishes following recession of innate immune responses. However, as the LV remodeling and progressive cardiac dysfunction progresses, CD4^+^ T-cells start transmigrating into the failing hearts again. Studies from us and others have shown that T-cells activated during these 2-phases (1–14 days post-MI and 8-week post-MI) are phenotypically and functionally different from each other. While, we have shown that TNF α (10) and estrogen receptor-α (11) expression are decreased in CD4^+^ T-cells early after MI, others have shown their cardioprotective nature and decreased pro-inflammatory characteristics meant to aid reparative macrophages (12). Our data further show that CD4^+^ T-cell activation during MI is restricted and their levels return to baseline by day 14. This is consistent with other studies showing that MHC-restricted CD4^+^ T-cells with TCR specificity against an immunogenic peptide of cardiac-specific myosin heavy chain-α preferentially accumulate in the mediastinal lymph nodes and the myocardium post-MI but subside rapidly and are not present during HF (12). However, during chronic HF (8-week post-MI) another cascade of CD4^+^ T-cell activation ensues which coincides with pathological LV remodeling and cardiac hypertrophy. Moreover, these HF-activated CD4^+^ T-cells are of pro-inflammatory phenotype (increased TNFα and IFNγ expression), and immunogenic as they infiltrate the myocardium upon adoptive transfer and induce cardiac dysfunction in naïve mice (4). Recent studies showing specificity against cardiac neoantigens (13) and the myocardium (4) as well as restricted TCR repertoire in cardiac CD4^+^ T-cells (12) lend further support to the presence of antigen-specific T-cells during HF. Our results showing ameliorated LV remodeling with temporal depletion of CD4^+^ T-cells also prove pathological behavior of CD4^+^ T-cells during chronic HF. Similar, detrimental CD4^+^ T-cell activation has also been shown in pressure-overload induced HF (5) and aging in mice (14). These findings signify the importance of elucidating detailed immune activation kinetics and temporal modulation of selective immune cell populations, rather than general immunosuppression, as a preferable strategy to derive therapeutic benefits during HF.

Innate immune responses exhibit phasic kinetics with predominance of phagocytic and pro-inflammatory immune cells in the myocardium for 1–4 days post-MI which is followed by the influx of anti-inflammatory, angiogenic, and profibrotic immune cells from 5 to 10 days to facilitate angiogenesis and scaring (1). These phasic responses were not observed with pro- and anti-inflammatory CD4^+^ T-cells, and all the major helper T-cell subsets viz Th1, Th2, Th17, and Tregs followed similar infiltration kinetics. Comparison of cell numbers also show that the cardiac milieu was predominated by Th1 and Tregs early after MI. This is consistent with prior studies showing increased Th1 T-cell levels post-MI and the need for Tregs to regulate their overt activation and to facilitate scar formation (2). Although, HF is also associated with increased levels of all T-cell subsets, we did not observe any predominance of pro- vs. anti-inflammatory subsets. However, considering the fact that global depletion of either all CD4^+^ T-cells or specific subsets, such as Tregs (8), blunt LV remodeling during HF suggest that during HF all CD4^+^ T-cells undergo a global pathological phenotypic shift. This change could be associated with T-cell anergy, or exhaustion as has been shown for CD8^+^ T-cells in other auto-immune diseases (15, 16). However, more detailed studies are warranted to identify key molecular mechanisms that lead to these pathological changes in CD4^+^ T-cells or to identify specific functional roles of helper T-cell subsets transmigrating into the failing hearts.

## Conclusion

MI and HF are immunologically different diseases, at least from the perspective of adaptive immune responses. Activation of CD4^+^ T-cells post-MI is a controlled response designed to subside rapidly with the scar formation to achieve complete immune resolution within 2 weeks post-MI. HF, on the other hand, is associated with a 2nd wave of CD4^+^ T-cell activation, and their transmigration into the heart promotes LV remodeling and progressive cardiac dysfunction. Temporal targeting of CD4^+^ T-cells could be an efficacious therapeutic modality for HF immunomodulation.

## Data availability statement

The original contributions presented in the study are included in the article/supplementary material, further inquiries can be directed to the corresponding author/s.

## Ethics statement

The animal study was reviewed and approved by the Institutional Animal Care and Use Committee at the Ohio State University.

## Author contributions

VK and SB conducted the experiments and analyzed the data. SP provided constructive feedback. SB designed all the studies and wrote the manuscript. All authors contributed to the article and approved the submitted version.

## Abbreviations

EDV: end-diastolic volume
EF: ejection fraction
ESV: end-systolic volume
HF: heart failure
LV: left-ventricle
MI: myocardial infarction
Th1, Th2 Th17: T helper cells 1/2/17
Tregs: regulatory T cells.
